# Green Tea (*Camellia sinensis*) Products as Alternatives to Antibiotics in Poultry Nutrition: A Review

**DOI:** 10.3390/antibiotics11050565

**Published:** 2022-04-23

**Authors:** Steve Kgotlelelo Mahlake, Caven Mguvane Mnisi, Cebisa Kumanda, Doctor Mziwenkosi Nhlanhla Mthiyane, Peter Kotsoana Montso

**Affiliations:** 1 Department of Animal Science, School of Agricultural Science, North-West University, Mafikeng 2745, South Africa; stevekgotlelelo@gmail.com (S.K.M.); doc.mthiyane@nwu.ac.za (D.M.N.M.); 2 Food Security and Safety Focus Area, Faculty of Natural and Agricultural Science, North-West University, Mafikeng 2745, South Africa; 21261660@nwu.ac.za; 3 Department of Animal Sciences, University of Pretoria, Pretoria 0002, South Africa; cebisakumanda@gmail.com; 4 Department of Microbiology, School of Biological Sciences, North-West University, Mafikeng 2745, South Africa

**Keywords:** antibiotics, meat quality, organic production, poultry, phytogenic plants

## Abstract

The overuse and misuse of antibiotics in poultry feeds increase the total cost of production and compromise the quality of poultry products, which poses a serious threat to human health. Globally, health-conscious poultry consumers have long called for the alternate use of natural additives to mitigate the development and spread of multidrug resistant pathogens. Phytogenic plants, such as green tea (*Camellia sinensis*) products, contain putative nutraceuticals with antibiotic properties that can be used as alternatives to therapeutic, metaphylactic, prophylactic, and growth-promoting antibiotics. However, there are limited studies in the literature that have evaluated the potential of green tea (GT) products when used as replacements to in-feed antibiotics, with most studies focusing on their potential as sources of dietary nutrients in poultry feeds. Thus, this review paper discusses the potential of GT products to replace various antibiotics in poultry diets while presenting GT bioactive substances that can improve the growth performance, carcass and meat quality traits, and health status of the birds. We postulate that the utilisation of GT products in place of antibiotics could deliver sustainable, organic poultry production systems that would contribute significantly to global food and nutrition security.

## 1. Introduction

High incidences of infectious disease outbreaks in the Global South contribute to the continuous usage of in-feed antibiotics as a strategy to reduce pathogenic microbial proliferation, foodborne diseases, and high mortality rates [1]. The presence of pathogenic microorganisms in the lower gastrointestinal tract (GIT) of poultry birds causes poor feed digestion and increases competition for dietary nutrients with the host and, thus, reduces growth performance. Consequently, various groups of antibiotics have been used to improve intestinal integrity, feed efficiency, growth performance, and to prevent or treat infectious diseases in food-producing animals [2]. However, the overuse of in-feed antibiotics in poultry diets has received wide criticism due to the development of endemic bacterial resistance and the occurrence of antibiotic residues in poultry products, which pose a serious threat to human health [3]. This has led to a shift in consumer preference towards antibiotic-free and organic products. From an economical viewpoint, the indiscriminate use of antibiotics by feed compounders increases production costs because synthetic antibiotics are unequivocally expensive [4]. In addition, the treatment of diseases caused by multidrug resistant pathogens may exacerbate the costs.

Although several countries around the world have banned the use of antibiotics, especially in food-producing animals, their complete withdrawal in disease-burdened areas without potential alternatives would inevitably result in poor productivity, resurgence in disease outbreaks, and the transmission of food-borne pathogens to humans. This will also have serious consequences on food and nutrition security [1]. Consequently, there is a need to identify and evaluate novel phytogenics that can be used in place of conventional antibiotics. Green tea (*Camellia sinensis*) products subscribe to such phytogenics with antioxidant, antimicrobial, gut-modulating, growth-stimulating, and health-promoting properties that can be used in poultry diets [5]. These products have been reported to have direct antimicrobial activity on *Streptococcus mutans* by inhibiting their attachment to oral mucosa [6].

Furthermore, GT products contain polyphenols (30% dry basis), such as flavanols, flavandiols, flavonoids, and phenolic acids, which are reported to exhibit antimicrobial effects against several bacterial species [6]. A recent study by Mahlake et al. [3] demonstrated that the inclusion of GT leaf powder in place of zinc-bacitracin antibiotic boosted the overall feed intake and carcass yield in adult Jumbo quail birds without compromising their health status. These findings could be attributed to the potential of GT catechins to inhibit microbial activities and improve gut health [7]. Although GT products have been incorporated in poultry diets [8], little is known about their effects as alternatives to conventional antibiotics. This review, therefore, discusses the potential of GT products to replace therapeutic, metaphylactic, prophylactic, and growth-promoting antibiotics in poultry diets while presenting GT bioactive substances that can be exploited to improve their growth performance, carcass and meat quality parameters, and the health status of the birds.

## 2. Use of Antibiotics in Poultry Production

The use of antibiotics in animal agriculture was initiated in the 1950s to respond to the increasing demand for food [9]. Since then, they have been used in animal production systems for treatment (therapeutic), control (metaphylaxis), or prevention (prophylaxis) of diseases [9] and for their non-medicinal purposes, such as growth promotion [10] to achieve economic gains [11]. Tetracycline, bacitracin, tylosin, salinomycin, virginiamycin, and bambermycin are the most commonly used antibiotics in intensive poultry production globally [12], with tetracyclines representing more than 66% of antimicrobials used in the United States of America (USA) [13] and only 37% in the European Union [14]. This high usage is attributed to the fact that tetracyclines are broad-spectrum antibiotics. In South Africa, tetracycline is also the most used antibiotic in livestock [15], with tylosin, sulphonamides, and penicillin being the most extensively sold [15]. Among these, tetracycline, penicillin, and sulphonamides are the most misused antibiotics in the country [16]. Whilst these antibiotics are beneficial to the poultry industry, their continuous usage causes environmental and ethical ordeals [17]. For this reason, European countries as well as New Zealand and the Korean Republic have outlawed their use in animal feeds. Similarly, other countries (Australia, Canada, Japan, and the USA) have enacted strict policies and regulations to control the use of antibiotics in food-producing animals [18]. Nonetheless, some large meat-producing countries such as Argentina, Brazil, China, India, Indonesia, the Philippines, Russia, and South Africa have not banned the use of antibiotic growth promoters (AGPs) [19]. As a result, multidrug resistant pathogens and antibiotic residues continue to occur in meat products, posing a serious threat both to the food industry and public health. This has fuelled growing consumer market interest in drug-free agricultural produce or organic products.

### 2.1. Therapeutic Antibiotics

Therapeutic antibiotics have been used in animals to treat clinical diseases and to promote the health and general welfare of animals [20,21]. According to Dibner and Richards [22], low and sub-therapeutic doses of antimicrobials play a vital role in the improvement of feed efficiency, promotion of animal growth, and prevention or control of diseases. However, these antibiotics are poorly utilised, and their residues may remain in animals’ bodies for a very long time. Tetracyclines are the most common antibiotic residues discovered in animal-derived foods (41%), followed by beta-lactams (18%) [23]. A three-year retrospective analysis of antimicrobial usage in cattle production in three states in Southwestern Nigeria indicated that tetracyclines, fluoroquinolones, and β-lactams were the most used antimicrobials [24]. Most livestock farmers use penicillin, tetracycline, fluoroquinolones, and amino glycosides with the main aim of preventing and treating infections [25]. While penicillin and tetracyclines are still more widely prescribed antibiotics for use in food-producing animals, macrolides, polymyxins, and aminoglycosides are also used in those animals. The antimicrobials routinely used as coccidiostats, such as ionophores and sulphonamides, are also used as prophylactics to prevent coccidiosis [11,26]. However, their continued misuse in animals may exacerbate the increasing trend towards the development of antimicrobial-resistant pathogens, antibiotic residues in meat products, and contamination of food [27]. This may not only compromise food safety, but it can also pose a health threat to humans. Against this backdrop, this has increased interest in the search for alternative therapies, including the use of safe and efficient natural phytotherapeutics, such as GT products. Indeed, GT polyphenols have the potential to work against poultry diseases, including avian influenza and coccidiosis [28].

### 2.2. Metaphylaxis Antibiotics

According to Bousquet-Melou et al. [29], the metaphylactic use of antibiotics refers to their administration to clinically healthy animals within a group demonstrating clinical signs. It is an early curative treatment initiated after the commencement of a disease but before the onset of clinical symptoms. Throughout the years, there has been a frequent usage of antibiotics in food-producing animals for non-therapeutic purposes more than for therapeutic applications [9]. Although many consumers feel that treating sick animals is appropriate, opinions on the use of antimicrobials in food-producing livestock are divided. Given that metaphylaxis reduces bovine respiratory disease, which is the most common cause of morbidity and mortality in beef cattle, livestock producers are concerned that eliminating such a widely used production technology would be harmful to animal health, resulting in significant animal deaths, reduced animal welfare, decreased production, and lower profitability. The use of metaphylactic antibiotics is more common in cattle feedlots, where tildipirosin and gamithromycin are used the most to prevent bovine respiratory diseases. In the USA, tilmicosin and ceftiofur have been phased out in cattle feedlots in favour of newer or other antimicrobials [30]. Even though tilmicosin and ceftiofur are being used in fewer feedlots for metaphylaxis purposes, the feedlot proportion on the use of tildipirosin and gamithromycin has increased. Since conventional antibiotics are non-selective, their repeated usage in animals for non-therapeutic purposes has been recognised as a contributing factor to the increasing development of antimicrobial resistance in foodborne pathogens [26]. In addition, antibiotics may induce the development of virulence and novel antimicrobial genes in non-pathogenic microbes. Thus, this prompted the need to use natural phytogenics possessing antimicrobial effects that can be used as alternatives to synthetic antibiotics. Green tea leaf meal has been shown to have anticoccidial activities against *Eimeria* parasites, which are responsible for causing economically important diseases such as coccidiosis [28].

### 2.3. Prophylaxis Antibiotics

Prophylaxis is a preventative measure that involves administering antibiotics to a flock of birds prior to the onset of a disease as a pre-emptive strategy to maintain health and avoid sickness [29]. Whilst the prophylactic use of antibiotics in chicken flocks is likely to cause enteric bacterial resistance with potential public health implications [31], there is currently limited empirical data showing the overall effects of prophylactic usage of antibiotics on poultry health. Notwithstanding the existing literature, early mass prophylactic antibiotic administration in layer chickens has been shown to cause a negative effect on adaptive immunity later in life [32]. Moreover, the prophylactic use of certain classes of antibiotics has been demonstrated to increase the risk of subsequent diarrhoea (tetracyclines, penicillins, lincosamides, and methenamines) and respiratory infections (penicillins, lincosamides, macrolides, and amphenicols) in commercial and small-scale indigenous chicken flocks in Vietnam [33]. This suggests that there is a need to explore natural additives that have the potential to boost the immune system and to prevent the occurrence of diseases. For example, GT phytochemicals have inhibitory effects on the growth of both Gram-negative and Gram-positive bacteria [34] and could assist in reducing the use of conventional antibiotics in birds.

### 2.4. Antibiotic Growth Promoters

Growth-promoting antibiotics refer to medicines administered at low subtherapeutic doses to destroy or inhibit bacterial growth [35]. Their use arose with livestock intensification in response to increased consumer demand for food [13]. In the poultry industry, they are used to improve the efficiency of growth, feed utilisation, and prevent diseases [36,37] and, therefore, are essential for successful economic development of poultry production [22]. They act against pathogens such as *Clostridium perfringens*, *Escherichia coli*, and *Salmonella* sp., which are found in the small intestines and are responsible for high mortality rates and poor meat quality in poultry [38]. Indeed, AGPs such as zinc bacitracin are largely used in poultry feeds because they possess antimicrobial properties against Gram-positive bacteria including *Staphylococcus*, *Clostridia*, and *Streptococcus* spp. [39]. Despite the threat they pose to human health, their use in poultry diets increases total production costs because they are expensive. Nevertheless, withdrawing the use of AGPs without potential replacements would cause bacterial infections to thrive, leading to reduced performance and disease outbreaks. It is, therefore, imperative to explore alternative natural products that have antimicrobial properties, such as green tea nutraceuticals [40]. Indeed, the total replacement of zinc bacitracin with GT powder increased overall feed intake and carcass performance in Jumbo quail [3]. The authors also reported that GT-containing diets had no negative effects on the health status of the birds.

## 3. Green Tea Products

Green tea (GT) is a perennial, cross-pollinated shrub with evergreen leaves, white blooms, and green fruits that was discovered by Emperor Shennong in China in 2737 BC [41]. The order *Ericales* and family *Theaceae* are both home to the green tea plant. Due to its health benefits, GT herbs are used to produce a variety of tea beverages for humans. Tea plants intended for human and animal consumption must be planted on good quality soil that is free of heavy metals, metalloids, and other pollutants, which are reported to cause morphological and physiological alterations, such as reduced growth rate, nutritional imbalance, and photosynthetic suppression [42]. The processing of the tea by fermentation is known to change its phenolic composition, with the concentration of some compounds increased after [43], whilst some compounds are reduced (e.g., theanine) [44].

There are two main methods used to manufacture tea, and these are crush-tear-curl (CTC) and orthodox. The CTC method uses a maceration device to produce the tea, whereas the orthodox method employs a roller or manual hand rolling [45,46]. Tea processing involves several steps depending on the final product. The initial step following harvest is withering, where the leaves are chopped and dried to reduce moisture content. After drying, the leaves are macerated to break cell walls and then fermented or deoxidised through enzymatic activities [45]. The polyphenol oxidase and peroxidase enzymes act aerobically on catechins to produce oxidised polyphenolic compounds such as theaflavins and thearubigins [46]. Moreover, the fermentation process enhances the quality of the tea. After the fermentation process, the product is dried to obtain the final tea product.

### 3.1. Green Tea Nutrients

**Figure 1:**
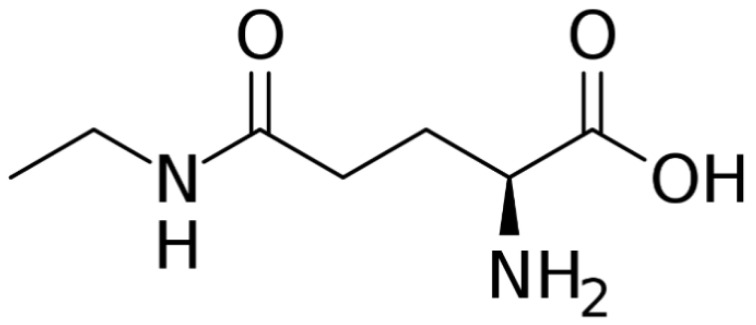
The structural formula of l-theanine [51].

Green tea products contain considerable amounts of important nutrients for poultry nutrition. The crude protein content of GT leaves ranges from 18.15% to 22.9% [47,48] and can meet the protein requirements of laying and breeding birds (20% CP) as recommended by the National Research Council [49]. In addition, the leaves contain varying amounts of amino acids, including methionine, threonine, leucine, and L-theanine (Figure 1) occurring in largest amounts. L-theanine (γ-glutamylethylamide) is a non-protein water-soluble amino acid that is the most abundant of all amino acids (~50%) in GT. It is reported to have pharmacological properties that are beneficial in poultry production [41], including serving as an antioxidant, growth promoter, immune booster, antimicrobial, and anti-inflammatory agent [50].

Abdo et al. [47] reported that GT leaves can provide between 11.3 and 14.6 MJ/kg metabolisable energy (Table 1), which is adequate for growing and laying quail (11.7 MJ/kg and 10.7 MJ/kg, respectively) [52]. The leaves also contain high concentrations of minerals and trace elements, vitamins (A, C, E, K, and B complex), lipids (α-linolenic and linoleic acids), and pigments (carotenoids and chlorophyll) [53], which could be the reason why most research focused on its potential as a source of dietary nutrients. Moreover, the leaves also possess complex carbohydrates, such as pectins, cellulose, hemicellulose, and lignin [54], which limit their utilisation in poultry, especially at higher dietary levels [55]. This necessitates the need to determine the optimum dietary inclusion level of GT products in each poultry species so that the wellbeing of the birds is not compromised [3].

### 3.2. Green Tea Bioactive Compounds

**Figure 2:**
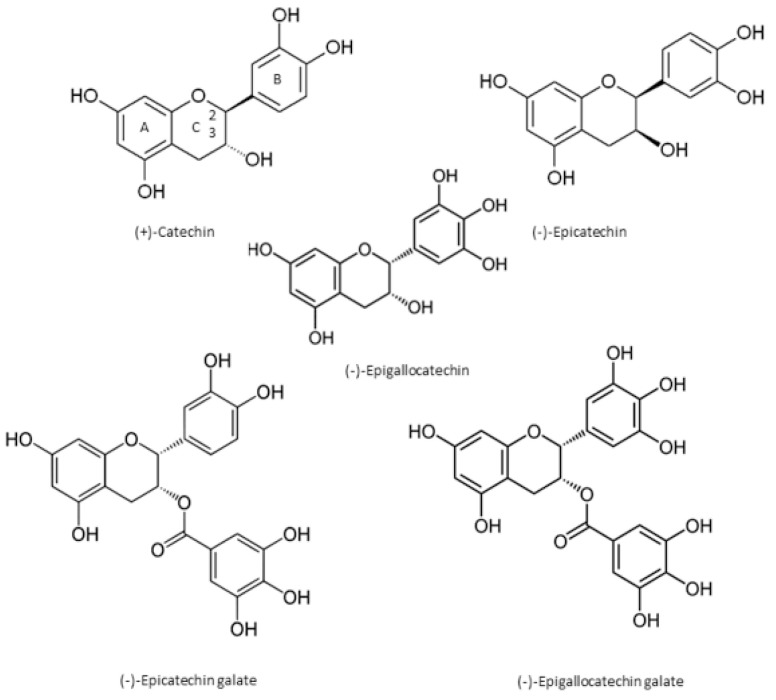
The structural formulae of catechins found in green tea leaves (Source: [67]).

Green tea is a rich source of biologically active compounds, such as catechins, epicatechins, l-theanine, theaflavins, flavonol glycosides (quercetin, kaempferol, and myricetin), theobromine (methylxanthine), caffeine, and volatile organic substances [57]. It is also a rich source of polyphenols, such as flavanols, flavandiols, flavonoids, and phenol acids, which have antioxidant and antimicrobial activities [47]. The antioxidant effects of GT are due to its ability to limit the number of free radicals by binding to reactive oxygen species [58]. Green tea catechins are the main polyphenols that are responsible for its health benefits linked to GT consumption [59]. Catechins are made up of three hydrocarbon rings and are classified into non-ester catechins (epicatechin (EC) and epigallocatechin (EGC)) as well as ester catechins (epicatechin gallate (ECG) and epigallocatechin gallate (EGCG)). Catechins such as EC, gallocatechin (GC), ECG, EGC, gallocatechin gallate (GCG), and EGCG [60] are monomeric units of condensed tannins with low molecular weights [59], as indicated in Figure 2. Farahat et al. [61] reported that GT leaves contain 0.7% catechin, 1.6% EC, 1.5% GC, 3.4% EGC, 7.15 EGCG, 3.8% ECG, and 1.6% GCG (*w*/*w*) polyphenolic components.

Generally, these compounds are colourless and water-soluble with a bitter and astringent taste. Green tea catechins have antimicrobial, antioxidant, hypoallergic, anticarcinogenic, and hypoglycaemic effects [62]. The most abundant catechin type found in GT is EGCG, which constitutes about 50 to 80% of total catechins in the leaves [63]. The effectiveness of antioxidant activities among GT catechins is greatest in EGCG, followed by ECG, EGC, and EC [64]. These polyphenols can help reduce the risks of stroke, cancer, and other cardiac-related diseases [65], and can reduce plasma and meat cholesterol in chickens [66].

The number and position of hydroxyl groups in the molecular structure of these bio-compounds influence their antioxidative effects such that catechins have a strong capacity to supply hydrogen on the B and C rings [68]. The unsaturated 4-oxo group and the 2,3-double bond in the C-ring stimulate the delocalization of electrons of the ortho-dihydroxy catechol in the B-ring [68]. In addition, the antioxidant action of these bioactive compounds is associated with molecular structure, microenvironment, and starting conditions of the reaction medium. Since all these compounds are made of a 4-oxo 3-hydroxy C ring structure, they possess oxidation resistance properties. Furthermore, GT consists of a wide range of antimicrobial activities due to the presence of catechins, particularly epigallocatechin gallate [69]. Hara [70] demonstrated the ability of GT polyphenols to effectively inhibit bacterial growth, especially *Vibrio cholera*, *Staphylococcus aureus*, and *Clostridium botulinum* species.

## 4. The Effect of Green Tea Products Inclusion in Poultry Diets

### 4.1. Effects on Nutrient Utilisation and Growth Performance

Green tea products can be used as alternatives to antibiotics to improve poultry performance and reduce enteric pathogenic microbial loads, thus improving nutrient digestion and absorption [71]. This is attributed to the presence of catechins, phenolic acids, and flavonols with antimicrobial, antioxidant, and anti-inflammatory effects [69]. These compounds can selectively alter gut microbiota through antimicrobial activity by affecting the survivability of microorganisms (mainly Gram-positive bacteria, *Eimeria* parasites, and avian influenza subtype viruses) through increased hydrophobicity [68]. This changes the characteristics of cell membranes causing ion leakage, thus making microbes less virulent [72] and resulting in improved feed efficiency, nutrient utilisation, and stimulation of the immune system. Improved nutrient utilisation is also a result of protection from the oxidative damage of lipids and improved absorption of nutrients in the gut [73]. Indeed, improvements in the feed conversion ratio, growth performance, and meat quality have been reported in broiler chickens reared on GT-containing diets [74]. Polyphenols such as catechins, phenolic acids, and flavanols found in GT have hypolipidemic, antimicrobial [75], and anticoccidial effects [28], which have been reported to improve the growth performance of broiler chickens [74]. Mahlake et al. [3] reported that replacing zinc-bacitracin antibiotic with GT leaf powder in Jumbo quail diets increased the overall feed intake but not the weight gain or feed conversion efficiency. This demonstrates the potential of green tea products to act as alternatives to in-feed antibiotics in poultry production.

### 4.2. Effects on Visceral Organs, Carcass, and Meat Quality Traits

In the interest of environmental sustainability and human health, the use of natural phytogenics like GT products in poultry nutrition is largely influenced by the demand for food products that are devoid of antibiotic residues. Indeed, Tuzcu et al. [76] reported that supplementing fattening quail diets with 200 or 400 mg/kg synthetic epigallocatechin gallate increased the carcass weight and dressing percentage under stress conditions (at 34 °C), which could be due to the antioxidant effects of GT polyphenols, as observed in previous studies with chickens [47,63,77]. Furthermore, the feeding of GT products to poultry birds has shown great potential to enhance the quality of meat for consumers. Kara et al. [78] observed a significant increase in water-holding capacity and antioxidant capacity whilst decreasing serum glucose and total cholesterol levels of breast meat in Japanese quail fed diets supplemented with 2.50 g/kg catechin. This improvement could be due to the catechins’ antioxidant effects that protect the cell walls against lipid peroxidation. It is evident that the use of GT products in poultry can improve carcass and meat quality parameters. This practice would encourage organic farming and production of poultry products that are free of antibiotics. Unfortunately, limited information is available regarding the effect of GT products as alternatives to antibiotics on carcass characteristics, meat quality, and stability traits.

### 4.3. Effects on Health Status of Poultry

Green tea bioactive compounds are known to improve immune responses in poultry birds by acting against *Eimeria* [28], a causative agent for coccidiosis, which is an economically important disease in the poultry industry. Infection by *Eimeria* destructs the intestinal epithelium cells, resulting in cell permeability, nutrient and plasma protein leakage, impaired nutrient absorption, and thus contributes to poor bird health [79]. Interestingly, supplementation of chicken diets with 5 and 20 g/kg GT products decreased *Eimeria maxima* faecal oocysts shedding by 38.5% and 51.5%, respectively, at five weeks of age [28]. Similarly, in vitro studies have reported that GT constituents have anti-parasitic activities by inhibiting egg hatching and larval development of *Trichostrongylus colubriformis* and *Teladorsagia circumcincta* [80]. Furthermore, Song et al. [69] studied catechin derivatives and their in vitro anti-influenza viral activity and reported inhibitory effects of the derivatives for avian influenza virus by inhibiting the absorption of the viruses in red blood cells.

The consumption of tea leaves has been shown to lower sugar uptake and reduce sugar levels in the blood by suppressing the glucose transporter activity in the intestinal epithelium in rats [81]. Green tea catechins also inhibit digestive lipases and interfere with the formation of lipid micelles in the intestine, resulting in lower fat absorption [82]. Kara et al. [83] further reported a decrease in serum triglyceride levels when layer quail diets were supplemented with 4 g/kg catechin. In addition, GT extracts can improve antibody responses against Newcastle disease virus [61], suggesting that GT bioactive compounds can be exploited to boost immunity in poultry birds [61].

### 4.4. The Effects on Gut Microbes

Microbial resistance to conventional antibiotics has become a global problem, which has led to the need to search for novel phytogenic plants as potent antimicrobial agents. A study by Zhao et al. [84] suggested that GT leaf meal polyphenols not only have antimicrobial effects on intestinal bacterial pathogens but also have effects on a wide range of viral and fungal pathogens. According to Zhao et al. [84], EGCG found in GT leaf meal is capable of cross-linking with proteins, causing damages to microbial cytoplasmic lipids and proteins, which results in a broad spectrum of antimicrobial activities. The main antibacterial mechanism of EGCG lies in its capacity to attach itself directly to the peptidoglycan layer of bacterial cell walls, causing damage to the cross-linking peptides and resulting in the destruction of cell walls [85]. Zhao et al. [84] also stated that EGCG damages bacterial cytoplasmic lipids, membrane proteins, or cytoplasmic enzymes, thus exerting antibacterial effects. As a result, EGCG weakens bacterial resistance to antibiotics and, in doing so, increases the sensitivity of bacteria to antibacterial agents. In an in vivo study by Bakkir et al. [86], the presence of green tea extract was associated with a reduction in the severity of necrosis and swelling in rabbits subcutaneously inoculated with methicillin resistant *S*. *aureus* (MSRA). Moreover, Al-Kayali et al. [87] reported that 10% aqueous GT extract and various antibiotics had similar antimicrobial effects against the antibiotic resistant *S*. *pyogenes*, *P*. *mirabilis,* and *S*. *aureus* species that cause diseases.

Gut microbiota play a vital role in poultry nutrition and health. They increase the digestion and absorption of nutrients, the supply of nutrients (secondary metabolites), as well as protect the GIT mucosa. Unlike conventional antibiotics, GT products may stimulate the growth of beneficial microbes while reducing pathogenic ones [88]. Catechins compounds have antimicrobial activities against both Gram-negative and Gram-positive bacteria [89]. For instance, studies have reported that GT could inhibit the synthesis of PBP2 protein in methicillin-resistant *Staphylococcus aureus* and Gram-negative bacteria [6,34]. The reduction in pathogenic microbes may decrease microbial competition in the GIT and, thus, enhance the growth of beneficial microbes. Moreover, GT products may contain other components (secondary metabolites) that can be utilised by beneficial microbes to synthesise their proteins, fatty acids, and vitamins.

## 5. Conclusions

High feed costs, microbial infestation, and oxidative stress remain the major constraints for sustainable poultry production. Moreover, the indiscriminate use of antibiotics to improve poultry performance and prevent or treat diseases constitutes a serious threat to human health. As such, phytogenics such as green tea products that contain essential nutrients and biologically active compounds with putative antibiotic activities should be evaluated for use as alternatives to conventional antibiotics. We conclude that the incorporation of green tea products in poultry diets as a replacement to antibiotics could deliver sustainable organic poultry production systems that would contribute significantly to global food and nutrition security.

## Figures and Tables

**Table 1:** Nutrient composition (%) of green and black tea leaf powders and their spent tea leaves.

Chemical Composition	Green Tea Powder	Black Tea Powder	Spent Green Tea Leaves	Spent Black Tea Leaves
Dry matter	90.7	94.2	13.4	12.6
Crude protein	22.9	24.2	24.6	23.4
Neutral detergent fibre	32.5	32.3	40.5	47.4
Acid detergent fibre	21.1	30.9	29.4	41.0
Organic matter	-	93.9	95.7	96.1
Ether extract	2.08	1.26	2.31	1.35
Tannin	22.3	-	-	-
Metabolizable energy (Kcal/kg)	2816	1529	1765	1574
Source	[48]	[56]	[56]	[56]

## Data Availability

Not applicable.
